# Robustness of sepsis-3 criteria in critically ill patients

**DOI:** 10.1186/s40560-019-0400-6

**Published:** 2019-08-29

**Authors:** Diana M. Verboom, Jos F. Frencken, David S. Y. Ong, Janneke Horn, Tom van der Poll, Marc J. M. Bonten, Olaf L. Cremer, Peter M. C. Klein Klouwenberg

**Affiliations:** 1 Julius Center for Health Sciences and Primary Care, University Medical Center Utrecht, Utrecht, the Netherlands; 2 Department of Intensive Care Medicine, University Medical Center Utrecht, Utrecht, the Netherlands; 3 Department of Medical Microbiology and Infection Control, Franciscus Gasthuis and Vlietland, Rotterdam, the Netherlands; 4 Department of Intensive Care Medicine, Amsterdam UMC, Academic Medical Center, University of Amsterdam, Amsterdam, the Netherlands; 5 Center for Experimental and Molecular Medicine, Amsterdam UMC, Academic Medical Center, University of Amsterdam, Amsterdam, the Netherlands; 6 Division of Infectious Diseases, Amsterdam UMC, Academic Medical Center, University of Amsterdam, Amsterdam, the Netherlands; 7 Department of Medical Microbiology, University Medical Center Utrecht, Utrecht, the Netherlands; 8 Department of Medical Microbiology and Immunology, Rijnstate Hospital, Arnhem, the Netherlands

**Keywords:** Sepsis, Septic shock, Incidence, Mortality, Critical care, Infection

## Abstract

**Background:**

Early recognition of sepsis is challenging, and diagnostic criteria have changed repeatedly. We assessed the robustness of sepsis-3 criteria in intensive care unit (ICU) patients.

**Methods:**

We studied the apparent incidence and associated mortality of sepsis-3 among patients who were prospectively enrolled in the Molecular Diagnosis and Risk Stratification of Sepsis (MARS) cohort in the Netherlands, and explored the effects of minor variations in the precise definition and timing of diagnostic criteria for organ failure.

**Results:**

Among 1081 patients with suspected infection upon ICU admission, 648 (60%) were considered to have sepsis according to prospective adjudication in the MARS study, whereas 976 (90%) met sepsis-3 criteria, yielding only 64% agreement at the individual patient level. Among 501 subjects developing ICU-acquired infection, these rates were 270 (54%) and 260 (52%), respectively (yielding 58% agreement). Hospital mortality was 234 (36%) vs 277 (28%) for those meeting MARS-sepsis or sepsis-3 criteria upon presentation (*p* < 0.001), and 121 (45%) vs 103 (40%) for those having sepsis onset in the ICU (*p* < 0.001). Minor variations in timing and interpretation of organ failure criteria had a considerable effect on the apparent prevalence of sepsis-3, which ranged from 68 to 96% among those with infection at admission, and from 22 to 99% among ICU-acquired cases.

**Conclusion:**

The sepsis-3 definition lacks robustness as well as discriminatory ability, since nearly all patients presenting to ICU with suspected infection fulfill its criteria. These should therefore be specified in greater detail, and applied more consistently, during future sepsis studies.

**Trial registration:**

The MARS study is registered at ClinicalTrials.gov (identifier NCT 01905033).

**Electronic supplementary material:**

The online version of this article (10.1186/s40560-019-0400-6) contains supplementary material, which is available to authorized users.

## Introduction

Sepsis is a life-threatening disease caused by a dysregulated host response to infection. Unfortunately, both early recognition and definitive confirmation of the diagnosis have proven to be difficult as sepsis is a very heterogeneous syndrome [1]. Since 1991, conceptual thinking about sepsis has focused on the presence of a systemic inflammatory response syndrome (SIRS). However, SIRS criteria are neither sensitive nor specific for infection and do not necessarily indicate a dysregulated or life-threatening host response [2, 3]. Furthermore, sepsis definitions that relied on SIRS criteria were highly sensitive to minor variations in frequency and timing, thereby affecting the reliability of the sepsis diagnosis [2].

Sepsis-3 definitions were developed to improve risk stratification among patients with a suspected infection, and their predictive validity regarding unfavorable clinical outcomes have been confirmed several times by now [4–12]. Rather than a systemic inflammatory response syndrome, these sepsis definitions require the development of organ failure during an infectious episode, which is operationalized by an increase in the Sequential Organ Failure Assessment (SOFA) score [13, 14]. Similarly, the septic shock-3 definition requires the presence of elevated serum lactate levels in addition to fluid-resistant hypotension [15].

Sepsis-3 definitions were also established to increase uniformity among reported incidence and mortality rates [13–15]. A consistent diagnosis of sepsis and septic shock between centers is particularly important for research and benchmarking purposes. Clinical data can be sensitive to different coding approaches, complicating comparisons of sepsis epidemiology among different cohorts [16, 17]. However, as only a little attention has been focused on the robustness of sepsis-3 criteria, we studied the effects of minor variations in the interpretation of the criteria on the incidence and related mortality of sepsis-3.

## Materials and methods

### Study design and population

This study was embedded within the Molecular Diagnosis and Risk Stratification of Sepsis (MARS) cohort [18]. Consecutive adult patients with newly suspected infection either upon presentation or during ICU stay were enrolled in two Dutch tertiary ICUs between June 2011 and April 2015 (University Medical Center Utrecht) or between June 2011 and January 2014 (Academic Medical Center Amsterdam).

Patients who had been admitted to another ICU for more than 1 day before transfer to one of the study centers were excluded, because information about possible previous infections and organ failures was not available. Patients who had been treated for an infection in the week prior to ICU admission and subsequently were admitted with a new infection were also excluded to avoid possible overlap between pre-existent and newly acquired organ failures. The institutional review board approved an opt-out consent procedure (protocol number 10-056C).

### Data and definitions

Trained researchers attended daily multidisciplinary rounds in the participating ICUs and prospectively recorded the presence of infection, SIRS, and organ failure [18, 19]. In this study, we use the terms “MARS-sepsis” and “MARS-shock” to indicate severe sepsis and septic shock according to prospective assessment of the presence of SIRS and organ failure, based on the 1991 and 2001 definitions of sepsis [20, 21] (see Table 1). The incidence and related mortality of MARS-sepsis are shown for illustrative purposes only and are not intended to provide a head to head comparison with sepsis-3 (which would have no clinical significance) nor to appraise the robustness of sepsis-3.

**Table 1:** Sepsis definitions

Old sepsis	
MARS-sepsis	Presence of ≥ 2 SIRS criteria and organ failure within a 4-day window around suspected infection^a, b^
MARS-septic shock	MARS-sepsis and use of vasopressor for hypotension within a 4-day window^a, c^
Sepsis-3	
Sepsis-3 (4-day window)	Suspected infection and an acute SOFA score increase of ≥ 2 points within a 4-day window ^a^
Septic shock-3	Sepsis-3 and vasopressor-dependent hypotension (i.e., circulatory SOFA score ≥ 2) plus an increased serum lactate level of > 2 mmol/L within a 4-day window ^a, d^
Assessments of minor variations in diagnostic criteria	
Reduced observation window	Similar to sepsis-3, but with a 2-day window around suspected infection (i.e., an increase between the day before and the day of the onset of infection)
Absolute SOFA score	Suspected infection and an absolute SOFA score of ≥ 2 points at the day of onset of infection and within a 4-day window^a^
Septic shock-3 ignoring lactate	Similar to septic shock-3, but without the requirement of increased serum lactate levels if not measured

*SIRS* = Systemic Inflammatory Response Syndrome, *SOFA* = Sequential Organ Failure Assessment

^a^ 4-day window = an observation window ranging from 2 days before the initiation of empirical antibiotics (onset of infection) until 1 day after the onset of infection

^b^ Organ failure for MARS-sepsis was defined as the following signs of organ hypoperfusion or dysfunction: areas of mottled skin; capillary refilling requiring 3 s or longer; urine output < 0.5 ml/kg for at least 6 h, > 1.5-fold elevated creatinine or renal replacement therapy; lactate > 2 mmol/l; abrupt change in mental status; abnormal electroencephalographic findings consistent with septic encephalopathy; platelet count < 100,000 platelets/ml or disseminated intravascular coagulation; acute respiratory distress syndrome and cardiac dysfunction, as defined by echocardiography or direct measurement of the cardiac index [22]”

^c^MARS-septic shock was defined as the use of norepinephrine in a dose of > 100 ng/kg/min for more than 50% of an observation day, dopamine > 5 mcg/kg/min or epinephrine for hypotension despite adequate fluid resuscitation (e.g., not including induced hypertension)

^d^Lactate was considered increased if it was increased once at any day during the 4-day time window

**Fig. 1:**
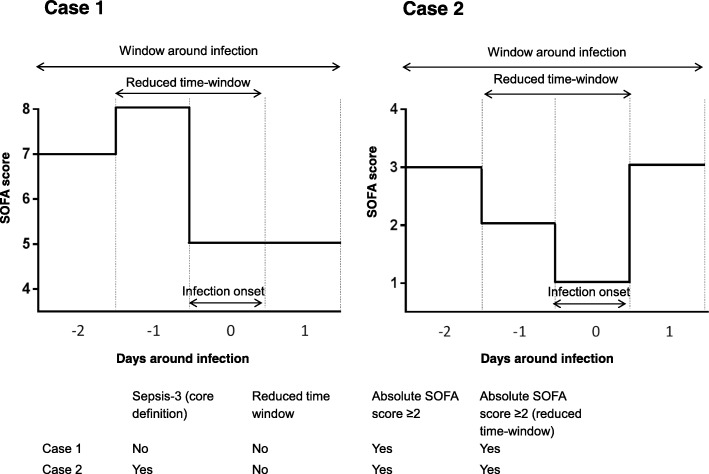
Hypothetical cases showing the influence of variations in organ failure definitions. SOFA = Sequential Organ Failure Assessment. The onset of infection (i.e., start of empirical antibiotic therapy) is day 0. Case 1 does not fulfill the sepsis-3 definition as there is no SOFA score increase of ≥ 2 points within the 4-day (or 2-day) time-window. However, case 1 fulfills the criteria if sepsis is defined by the presence of an absolute SOFA score of ≥ 2 (both in the 4-day and 2-day time-window). Case 2 fulfills the sepsis-3 criteria since there is an increase of ≥ 2 points between day 0 and day 1. In a reduced time-window, there is no increase observed between the day before infection and day of the onset of infection, and sepsis-3 criteria are not met

The terms “sepsis-3” and “septic shock-3” were used to indicate events meeting the updated definitions. Organ failure for sepsis-3 was defined as life-threatening organ dysfunction caused by a dysregulated host response to infection [14]. We operationalized organ failure as an acute SOFA score increase of ≥ 2 points compared to pre-existing (acute or chronic) organ dysfunction before the onset of infection (Table 1). The increase in SOFA score had to occur between 2 days before the onset of infection and 1 day after the onset of infection (i.e., a 4-day window, see Fig. 1). This window was used because organ dysfunction may occur prior to, near the moment, or after the infection is recognized [5]. An infection was registered when empirical antimicrobial therapy was started by attending clinicians irrespective of the presence of SIRS or organ failure, and this day was regarded as its onset. Subsequently, the likelihood of each infection was subsequently adjudicated as none, possible, probable or definite, using detailed definitions derived from Center of Disease Control and International Sepsis Forum Consensus Conference criteria [18, 23, 24]. Only first ICU infections occurring during a hospital admission were included for analysis. Infections present at admission (having onset between 1 day before and 2 days after ICU admittance) and ICU-acquired infections (having onset more than 2 days after ICU admittance) were analyzed separately since we hypothesized that the extent of new organ failure might vary between these types of infection.

To reconstruct baseline SOFA scores, raw pre-ICU clinical data were extracted from the hospital electronic health care record. All ICU data were collected prospectively [19]. In cases on dialysis dependency or having chronic renal insufficiency, the renal SOFA was assumed to be 3.

To evaluate the robustness, we assessed the influence of minor variations in the implementation of the sepsis-3 definitions (see Table 1). We based our variations on the methodology that was used in previous studies [4, 6, 13, 15]. First, we shortened the time window of observation by only including the day of clinical diagnosis and 1 day before (2-day window). Second, we explored the effects of an absolute SOFA score at the time of recognition of infection. Third, to mimic settings in which lactate is not always available, only vasopressor-dependent hypotension was required to fulfill the septic shock definition in cases where lactate levels were missing (see Table 1 and Fig. 1 for further explanations).

### Statistical analyses

We calculated apparent incidences and related in-hospital mortality of sepsis-3 and MARS-sepsis. We calculated the percent agreement as the percentage of cases in which two sepsis definitions corresponded with each other. Sensitivity analysis was performed by excluding subjects with rejected infection (i.e., a post hoc likelihood of none). All analyses were performed and reported separately for infections at admission and ICU-acquired infections. Missing data were handled as described in Additional file 1: Table S1. Differences at baseline and clinical characteristics between the subgroups were analyzed using a Mann-Whitney *U* test, chi-square test, or McNemar test, as appropriate. Differences in mortality were calculated accounting for partially overlapping samples [25]. A *p* value < 0.05 was considered statistically significant. All analyses were performed using SAS 9.2 (SAS Institute Inc.).

## Results

**Fig. 2:**
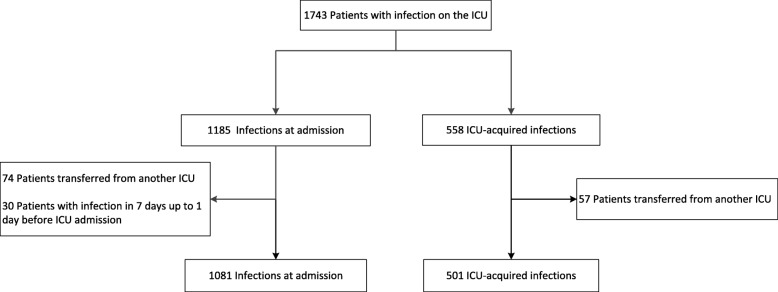
Flowchart. ICU = intensive care unit

**Fig. 3:**
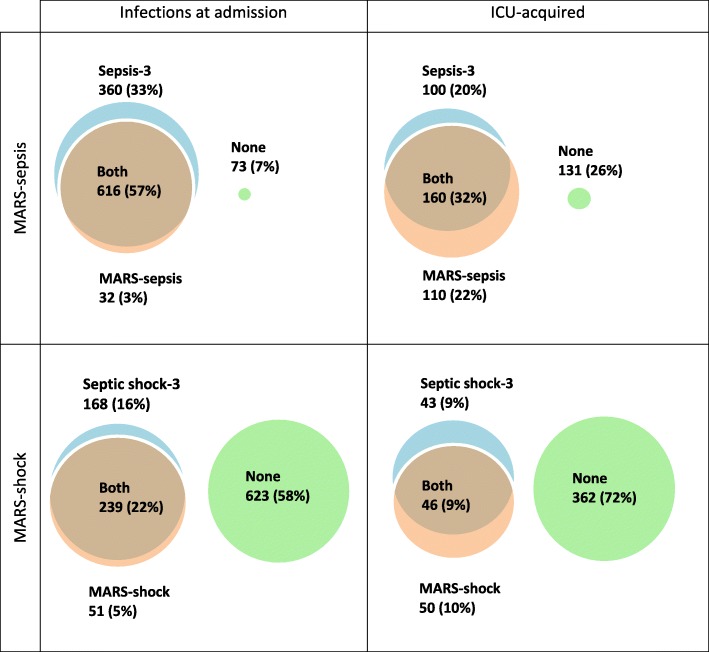
Venn diagram comparing MARS-sepsis and sepsis-3 definitions. ICU = intensive care unit. Presented as frequencies of patients (%)

Among 1743 patients treated for an infection in the ICU, 1081 with an infection at ICU admission and 501 with an ICU-acquired infection remained for analysis (Figs. 2 and 3). Patient and infection characteristics are presented in Table 2.

**Table 2:** Characteristics of patients with infection on admission and with ICU-acquired infection and stratified by presence of sepsis-3 criteria

	Infection at admission (*N* = 1081)			ICU-acquired infection (*N* = 501)		
	No sepsis-3 (*N* = 105)	Sepsis-3 (*N* = 976)	*p* value	No sepsis-3 (*N* = 241)	Sepsis-3 (*N* = 260)	*p* value
Age (years)	61 (42, 69)	64 (53, 73)	0.005	62 (51, 71)	61 (50, 71)	0.64
Male	64 (61%)	621 (64%)	0.59	175 (73%)	177 (68%)	0.27
Charlson comorbidity index	0 (0, 2)	1 (0, 2)	0.002	0 (0, 2)	0 (0, 2)	0.33
Chronic renal insufficiency^a^	9 (9%)	114 (12%)	0.34	20 (8%)	25 (10%)	0.6
APACHE IV Score	69 (50, 89)	83 (66, 03)	0.001	76 (58, 95)	76 (62, 99)	0.24
Medical admission	69 (66%)	726 (74%)	0.06	102 (42%)	104 (40%)	0.60
At onset of infection						
Days from ICU admission	0 (0, 2)	0 (0, 0)	< 0.001	6 (4, 8)	6 (4, 9)	0.32
Hospital-acquired infection	65 (62%)	449 (46%)	0.002	100%	(100%)	–
Vasopressor use	36 (35%)	663 (68%)	< 0.001	98 (41%)	141 (54%)	0.002
Mechanical ventilation	78 (74%)	664 (68%)	0.19	189 (78%)	240 (92%)	< 0.001
Lactate measured	37 (35%)	676 (69%)	< 0.001	72 (30%)	109 (42%)	0.005
Lactate	1.8 (1, 4)	3 (2, 5)	0.002	2 (1, 2)	2 (2, 4)	< 0.001
≥ 2 SIRS criteria	88(83%)	900 (92%)	0.004	205 (85%)	226 (87%)	0.55
SOFA score	2 (1, 4)	6 (4, 9)	< 0.001	6 (4, 8)	8 (5, 10)	< 0.001
Source of infection			0.08			0.39
Pulmonary tract	70 (67%)	533 (55%)		138 (57%)	154 (59%)	
Abdominal tract	7 (7%)	156 (16%)		7 (3%)	12 (5%)	
Urinary tract	6 (6%)	57 (6%)		1 (0%)	1 (0%)	
CRBSI	1 (1%)	15 (2%)		36 (15%)	25 (10%)	
Other	21 (20%)	215 (22%)		59 (24%)	68 (26%)	
Infection likelihood			0.02			0.13
▪ None	11 (10%)	99 (10%)		85 (35%)	82 (32%)	
▪ Possible	46 (44%)	298 (31%)		109 (45%)	105 (40%)	
▪ Probable	30 (29%)	293 (30%)		31 (13%)	43 (17%)	
▪ Definite	18 (17%)	286 (29%)		16 (7%)	30 (12%)	
Outcome						
Length of ICU stay (days)	2 (1, 6)	4.0 (2, 10)	< .001	6 (3, 13)	7 (3, 15)	0.12
Length of hospital (days)	13 (5, 29)	15 (7, 31)	0.09	19 (9, 34)	22 (9, 38)	0.64
ICU mortality	8 (8%)	197 (20%)	0.002	52 (22%)	82 (32%)	0.01
Hospital mortality	12 (11%)	277 (28%)	0.001	72 (30%)	103 (40%)	0.05
90-day mortality	20 (19%)	328 (34%)	0.002	83 (35%)	114 (44%)	0.03

*APACHE* = acute physiologic and chronic health evaluation, *SIRS* = systemic inflammatory response syndrome, *ICU* = intensive care unit, *CRBSI* = catheter-related bloodstream infection. Continuous data are presented as medians (IQR), dichotomous data are presented as frequencies (%)

^a^Creatinine >170 mmol/L or dialysis dependency

### Incidence and associated mortality

Table 3 shows the apparent incidences and related percent agreement of sepsis and septic shock according to the various definitions.

**Table 3:** Incidences of sepsis and related mortality according to core definitions

	*N*	Sepsis-3 incidence, % (95%CI)	MARS-sepsis incidence, % (95%CI)	Agreement (%)	*p* value ^a^	Sepsis-3 mortality, % (95%CI) ^b^	MARS-sepsis mortality, % (95%CI) ^b^	*p* value ^a^
Complete cohort	1582							
▪ Infection at admission	1081	90 (88–92)	60 (57–63)	64	< 0.001	28 (26–31)	36. (33–40)	< 0.001
▪ ICU-acquired infection	501	52 (48–56)	54 (50–58)	58	0.49	40 (34–46)	45 (39–51)	< 0.001
Probable infection cohort ^c^	1304							
▪ Infection at admission ^c^	971	90 (88–92)	61 (58–64)	65	< 0.001	29 (26–32)	37 (33–41)	< 0.001
▪ ICU-acquired infection ^c^	334	53 (48–59)	56 (51–62)	59	0.39	44 (37–51)	51 (42–56)	< 0.001
		Septic shock-3 incidence, % (95%CI) ^a^	MARS-shock incidence, % (95%CI) ^a^	Agreement (%)	*p* value	Septic shock-3 mortality, % (95%CI) ^b^	MARS-shock mortality, % (95%CI) ^b^	
	*N*							
Complete cohort	1582							
▪ Infection at admission	1081	38 (35–41)	27 (24–30)	80	< 0.001	41 (36–46)	50 (45–56)	< 0.001
▪ ICU-acquired infection	501	18 (15–21)	19 (16–23)	81	0.47	57 (47–67)	69 (59–78)	< 0.001
Probable infection cohort ^c^	1304							
▪ Infection at admission ^c^	971	39(36–42)	28 (25–31)	79	< 0.001	42 (37–47)	51 (46–57)	< 0.001
▪ ICU-acquired infection ^c^	334	19 (15–23)	22 (18–27)	83	0.11	63 (50–74)	73 (62–82)	< 0.001

ICU=intensive care unit

^a^ McNemar test comparing the incidence of sepsis-3 and MARS definitions

^b^ Mortality reflects in-hospital mortality. For all definitions, mortality of the sepsis-3 criteria was significantly lower than mortality of the MARS definitions (*p* < 0.001)

^c^A subgroup of patients in whom the infection diagnosis was either possible, probable or definite based on microbiology, clinical symptoms, and radiology, as defined by post hoc assessment

Compared to prospectively recorded MARS-sepsis events, more patients fulfilled sepsis-3 and septic shock-3 criteria at ICU admission (60% vs 90%, and 27% vs 38%, respectively). Furthermore, agreement between the definitions was only 64% and 80%, respectively. For patients with ICU-acquired infections, the overall incidences of sepsis (54% vs 52%) and septic shock (19% vs 18%) were similar, yet the MARS and sepsis-3 criteria selected different individuals (58% and 81% agreement for sepsis and septic shock, respectively) (Table 3).

Hospital mortality was lower for patients with sepsis-3 and septic shock-3 than for patients with MARS-sepsis and MARS-shock (Table 3). Indeed, those patients who were exclusively identified by sepsis-3 at admission (33% of all patients) had a lower mortality rate than patients with organ failure according to both MARS-sepsis and sepsis-3 (37% vs 14%, respectively) (Additional file 1: Table S2). Nevertheless, mortality was > 10% for all definitions (Table 3, Additional file 1: Table S2 and Table S3). There were 110 (10%) and 167 (33%) patients with a rejected infection (i.e., those with a post hoc likelihood rated as none) at ICU admittance and during admission respectively. The exclusion of patients with rejected infection had a negligible effect on apparent sepsis incidences, mortality, and agreement (Table 3).

### Robustness of the sepsis-3 definitions

Table 4 shows the results of the analyses to assess the robustness of sepsis-3 criteria. Minor variations in the timing of observations and criteria for organ failure considerably affected the apparent incidence of sepsis-3 at admission, ranging from 68 to 96% for the most restrictive and the most liberal definition, respectively. Using the same criteria, the incidence of septic shock-3 varied from 30 to 42%. For ICU-acquired infections, the incidence of sepsis-3 and septic shock-3 ranged from 22 to 99% and from 7 to 28%, respectively. Whereas these minor variations did not affect hospital mortality rates for infections at admission, and only marginally for ICU-acquired sepsis (Table 4).

**Table 4:** The influence of minor variations in diagnostic criteria on the apparent incidence and related mortality of sepsis

Core definitions and minor variations	Incidence, % (95%CI)	Agreement (%) ^a^	*p* value ^b^	Mortality, % (95%CI)	*p* value
Infection at admission					
Sepsis-3					
▪ Core definition: SOFA increase (4-day window)	90 (88–92)	n/a	n/a	28 (26–31)	n/a
▪ SOFA increase (2-day window)	68 (66–71)	78	< 0.001	28 (25–31)	0.50
▪ Absolute SOFA ≥ 2 (4-day window)	96 (95–97)	94	< 0.001	27 (25–30)	0.45
▪ Absolute SOFA ≥ 2 at onset of infection	89 (87–91)	88	0.30	28 (25–31)	0.64
Septic shock-3					
▪ Core definition: SOFA increase (4-day window)	38 (35–41)	n/a	n/a	41 (36–46)	–
▪ SOFA increase (2-day window)	30 (27–32)	92	< 0.001	41 (36–46)	0.98
▪ Absolute SOFA ≥ 2 (4-day window)	39 (36–42)	99	< 0.001	41 (36–45)	0.81
▪ Absolute SOFA ≥ 2 at onset of infection	37 (34–40)	97	0.25	41 (37–46)	0.83
▪ Shock-3 ignoring lactate	42 (40–45)	95	< 0.001	41 (36–45)	0.93
ICU-acquired infection					
Sepsis-3					
▪ Core definition: SOFA increase (4-day window)	52 (48–56)	n/a	n/a	40 (34–46)	n/a
▪ SOFA increase (2-day window)	22 (19–26)	70	< 0.001	42 (33–51)	0.31
▪ Absolute SOFA ≥ 2 (4-day window)	99 (97–100)	53	< 0.001	35 (31–39)	<0.01
▪ Absolute SOFA ≥ 2 at onset of infection	96 (94–98)	53	< 0.001	35 (31–40)	<0.01
Septic-shock-3					
▪ Core definition: SOFA increase (4-day window)	18 (15–21)	n/a	n/a	57 (47–67)	n/a
▪ SOFA increase (2-day window)	7 (5–9)	89	< 0.001	65 (48–79)	0.05
▪ Absolute SOFA ≥ 2 (4-day window)	27 (23–31)	91	< 0.001	54 (45–62)	0.26
▪ Absolute SOFA ≥ 2 at onset of infection	26 (22–30)	90	< 0.001	53 (44–61)	0.14
▪ Shock-3 ignoring lactate	28 (24–32)	90	< 0.001	50 (42–58)	0.01

*SOFA* = Sequential Organ Failure Assessment. Incidences are the apparent incidences of the various sepsis-3 variations

^a^Percentage agreement indicates the agreement of the incidence with the incidence of the core definition (≥ 2 increase in SOFA score) of sepsis-3

^b^McNemar test comparing the incidence of the core definition and minor definitions

## Discussion

We assessed the incidence, mortality, and robustness of the sepsis-3 definitions in a large prospectively monitored cohort of ICU patients. We found that virtually all patients with a suspected infection met clinical criteria for organ failure and, as such, the sepsis-3 criteria did not have discriminative power in our setting. Furthermore, minor variations in the precise interpretation of the criteria required to meet the sepsis-3 definitions considerably impacted the apparent incidences of both sepsis and septic shock, while mortality remained comparable among the variations.

An anticipated advantage of the sepsis-3 definitions is that they may increase the comparability of sepsis incidence and related mortality among studies. Organ failure is explicitly defined by means of the SOFA score, possibly reducing subjective interpretation. Still, studies published to date have used many subtle variations on the original definition. For example, the original publication suggested to define organ failure as an acute change in the SOFA score of ≥ 2 points as a consequence of infection [14]. Subsequent validation studies, however, have largely disregarded this requirement of an acute SOFA increase. Instead, they used an absolute SOFA score of ≥ 2 points, applied different time-windows, and used different ways of taking chronic comorbidities into account [4–10, 13]. By applying similar (minor) variations to our data, we explored the robustness of the criteria and observed considerable variations in the apparent incidences of sepsis-3. Similar variations in the incidence of sepsis-3 and septic shock-3 are likely to occur in other studies, hence affecting the comparability of study results. Standardization of the operationalization of sepsis-3 criteria is therefore paramount to improve the generalizability of studies.

One of the most used and straightforward methods of defining organ failure for sepsis diagnosis is the use of an absolute SOFA score, thereby disregarding any pre-existent organ failure. And yet, several problems might arise using this approach. First, almost all ICU patients fulfill these criteria, indicating that the criteria have no discriminatory power in ICU settings. Second, an absolute SOFA score disregards the etiology of organ failure. Organ failure might have been present already before infection (e.g., due to non-infectious diseases or pre-existent comorbidities) and is therefore not caused by the infection itself. To illustrate, in the current study, up to 33% of the patients who developed sepsis-3 actually did not have an infection in a post hoc adjudication. It therefore remains essential to differentiate between infectious and non-infectious causes for organ failure. We find that future efforts should also be directed to improve (risk) stratification of septic patients and enrich classification by inclusion of additional variables, such as type of organ failure, number of different organ dysfunctions, site of infection, and possibly biomarkers [26].

Our study has some limitations. First, organ failure data were often missing before ICU admission, which was also noticed in the original assessment of sepsis-3 [13]. Second, we based our severe sepsis and septic shock definitions on consensus literature. Nevertheless, the exact application of the definitions in our study might be different from others. Of note, some of the described restraints of the sepsis-3 criteria also apply to previous sepsis definitions.

## Conclusions

Virtually all patients who have suspected infection upon presentation to the ICU meet sepsis-3 criteria, making this definition less suitable for risk stratification in this setting. Furthermore, caution should be taken when using the sepsis-3 definitions to report incidences and related outcomes of sepsis, as they are very sensitive to minor variations in timing and interpretation of organ failure criteria. These criteria should therefore be specified in great detail, and applied very consistently, in all future publications on the topic.

## Additional file

Additional file 1: **Table S1.** Missing data. **Table S2.** Incidence, organ failure, and mortality of sepsis-3 and MARS-sepsis. **Table S3.** Incidence, organ failure, and mortality of septic shock-3 and MARS-shock. (PDF 40 kb)

## Data Availability

The datasets generated and analyzed during the current study are not publicly available. An extract can be available from the corresponding author on reasonable request.

## Abbreviations

APACHE: Acute Physiology and Chronic Health Evaluation
CI: Confidence interval
ICU: Intensive care unit
IQR: Interquartile range
MARS: Molecular Diagnosis and Risk Stratification of Sepsis
SIRS: Systemic Inflammatory Response Syndrome
SOFA: Sequential Organ Failure Assessment
