# Supplementary material for: Robustness of sepsis-3 criteria in critically ill patients
Source: J Intensive Care. 2019 Aug 29;7:46. doi: 10.1186/s40560-019-0400-6 (PMC6716896; doi:10.1186/s40560-019-0400-6)
Supplement: Supplementary file 1 — Table S1. Missing data. Table S2. Incidence, organ failure, and mortality of sepsis-3 and MARS-sepsis. Table S3. Incidence, organ failure, and mortality of septic shock-3 and MARS-shock. (PDF 40 kb) [file 40560_2019_400_MOESM1_ESM.pdf]

Additional file 1.

Robustness of sepsis-3 criteria in critically ill patients

Diana M. Verboom *et al.*

Table S1. Missing data

Variable	Pre-ICU days	Days on ICU
Number of days	1100	4057
All SOFA components missing	2.5%	0.0%
5 SOFA components missing	14.3%	0.9%
2-4 SOFA components missing	71.6%	0.1%
1 SOFA component missing	10.3%	0.1%
No SOFA components missing	1.5%	98.8%
Missing data by SOFA score component[a]		
Central nervous system	81.4%	0.1%
Circulatory	13.4%	1.0%
Renal	24.8%	0.9%
Respiratory	58.6%	1.1%
Liver	77.6%	1.1%
Coagulation	47.2%	1.0%
Missing data of other variables		
Serum lactate	81.3%	45.6%

ICU=intensive care unit, SOFA=Sequential Organ Failure Assessment.

[a] SOFA components were assumed to be normal when missing, except for renal scores. The renal SOFA score was assumed to be 3 in case of dialysis dependency and chronic renal insufficiency (creatinine > 170 mmol/L).

Table S2. Incidence, organ failure, and mortality of sepsis-3 and MARS-sepsis

S2a. Incidence

Infection at admission			
		MARS organ failure	
		Yes	No
Sepsis-3	Yes	616 (57%)	360 (33%)
organ failure	No	32 (3%)	73 (7%)
ICU-acquired infection			
		MARS organ failure	
		Yes	No
Sepsis-3	Yes	160 (32%)	100 (20%)
organ failure	No	110 (22%)	131 (26%)

S2b. Median SOFA scores at onset of infection

Infection at admission			
		MARS organ failure	
		Yes	No
Sepsis-3	Yes	8 (5-10)[a]	4 (3-6)[a]
organ failure	No	4 (3-9)	1 (1-3)
ICU-acquired infection			
		MARS organ failure	
		Yes	No
Sepsis-3	Yes	9 (7-12)[a]	6 (4-7)[a]
organ failure	No	3 (2-8)	5 (3-7)

S2c. Hospital mortality

Infection at admission			
		MARS organ failure	
		Yes	No
Sepsis-3	Yes	228 (37%)[b]	49 (14%)[b]
organ failure	No	6 (19%)	6 (8%)
ICU-acquired infection			
		MARS organ failure	
		Yes	No
Sepsis-3	Yes	77 (48%)[b]	26 (26%)[b]
organ failure	No	44 (40%)	28 (22%)

ICU=intensive care unit, SOFA=Sequential Organ Failure Assessment Data are presented as frequencies (%) and SOFA score as median (IQR).

[a]Organ failure was significantly lower in patients with sepsis-3 than for patients with both types of organ failure for both infection at admission and ICU-acquired infection ($p<0.0001$).

[b]Hospital mortality of patients with organ failure according to sepsis-3 was significantly lower than for patients with both types of organ failure for both infection at admission and ICU-acquired infection ($p<0.0001$).

Table S3. Incidence, organ failure, and mortality of septic shock-3 and MARS-shock

S3a. Incidence

		MARS-shock	
Infection at admission			
		Yes	No
Septic shock-3	Yes	239 (22%)	168 (16%)
	No	51 (5%)	623 (58%)
ICU-acquired infection			
		Yes	No
Septic shock-3	Yes	46 (9%)	43 (9%)
	No	50 (10%)	362 (72%)

S3b. Median SOFA scores at onset of infection

		MARS-shock	
Infection at admission			
		Yes	No
Septic shock-3	Yes	10 (7-12)[a]	8 (5-10)[a]
	No	9 (6-11)	4 (2-6)
ICU-acquired infection			
		Yes	No
Septic shock-3	Yes	12 (9-15) [a]	9 (7-12)[a]
	No	11 (8-13)	6 (4-8)

S3c. Hospital mortality

		MARS-shock	
Infection at admission			
		Yes	No
Septic shock-3	Yes	128 (54%)[b]	38 (23%)[b]
	No	18 (35%)	105 (17%)
ICU-acquired infection			
		Yes	No
Septic shock-3	Yes	33 (72%)[b]	18 (42%)[b]
	No	33 (66%)	91 (25%)

ICU=intensive care unit, SOFA=Sequential Organ Failure Assessment Data are presented as frequencies (%) and SOFA score as median (IQR).

[a]Organ failure was significantly lower in patients with septic shock-3 than for patients with both types of septic shock for both infection at admission and ICU-acquired infection ($p<0.01$).

[b] Hospital mortality of patients with septic shock according to sepsis-3 was significantly lower than for patients with both types of septic shock for both infection at admission and ICU-acquired infection ($p<0.01$).
